# FoxO1 Responses to Chronic Oxidative Stress to Participate in Age‐Related Osteoporosis by Depriving β‐Catenin From TCF7

**DOI:** 10.1111/acel.70306

**Published:** 2025-12-09

**Authors:** Peihong Su, Xiaoli Ma, Chong Yin, Ruilin Shi, Siyu Chen, Lihuizi Yang, Meng Qu, Xinyao Jia, Qi Yu, Hui Li, Airong Qian, Ye Tian

**Affiliations:** ^1^ Lab for Bone Metabolism, Xi'an Key Laboratory of Special Medicine and Health Engineering, Key Lab for Space Biosciences and Biotechnology, Research Center for Special Medicine and Health Systems Engineering, NPU‐UAB Joint Laboratory for Bone Metabolism, School of Life Science and Technology Northwestern Polytechnical University Xi'an Shaanxi China; ^2^ Shaanxi Provincial Key Laboratory of Ischemic Cardiovascular Disease Institute of Basic and Translational Medicine, Xi'an Medical University Xi'an Shaanxi China; ^3^ Department of Laboratory Medicine Translational Medicine Research Center, North Sichuan Medical College Nanchong China; ^4^ Department of Adult Joint Reconstruction Hong‐hui Hospital, Xi'an Jiaotong University Xi'an Shaanxi China; ^5^ Shenzhen Research Institute of Northwestern Polytechnical University Shenzhen China

**Keywords:** age‐related osteoporosis, FoxO1, MACF1, osteoblastic cell, ROS

## Abstract

The increasing prevalence of age‐related osteoporosis has emerged as a critical public health issue in the context of the globally aging population. Chronic oxidative stress, induced by excessive reactive oxygen species (ROS) associated with aging, is a critical factor underlying the development of osteoporosis in elderly individuals and a diminished capacity for bone formation and osteogenic differentiation. However, the mechanism underlying age‐related osteoporosis remains unclear. MACF1 (microtubule actin crosslinking factor 1) is an essential factor that regulates bone formation and development, and exhibits reduced expression as humans age. In this study, we used MACF1 conditional knockout (MACF1‐cKO) mice as a premature aging model and found that MACF1‐cKO mice exhibited chronic oxidative stress. Moreover, the expression level, nuclear translocation, and transcriptional activity of FoxO1 were promoted in MACF1 deficient osteoblastic cells. In addition, the binding of FoxO1 to β‐catenin was enhanced, increasing the transcriptional activity of the FoxO1/β‐catenin pathway in MACF1 deficient osteoblastic cells. The enhanced FoxO1/β‐catenin pathway competitively weakens the binding of β‐catenin to TCF7 and decreases the activity of the TCF7/β‐catenin pathway. Our study showed that FoxO1 responded to chronic oxidative stress induced by MACF1 deficiency to determine β‐catenin fate and regulate osteoblast differentiation during senile osteoporosis.

## Graphical abstract

Chronic oxidative stress caused by MACF1 deficiency leads to a functional switch in osteoblasts from “osteogenic differentiation” to “antioxidation.” FoxO1 responds to oxidative stress by altering the destination of β‐catenin and disrupting the balance of the FoxO1/β‐catenin–Wnt/β‐catenin pathway, thereby inhibiting osteoblast differentiation.

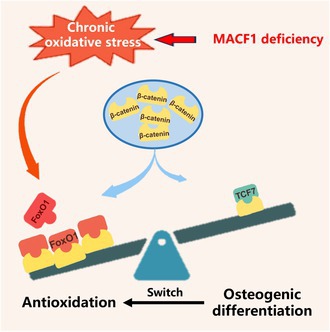

## 1 Introduction

Aging‐related osteoporosis is a prevalent condition characterized by decreased bone mass and deterioration of bone microarchitecture, leading to an increased risk of fracture (Hendrickx et al. 2015). As the human population ages, the occurrence of age‐related osteoporosis increases, posing a significant health threat to older adults. The etiology of age‐related osteoporosis is multifaceted and complex. Studies have shown that osteogenic differentiation is impeded by various signaling pathways during the aging process in both males and postmenopausal females, resulting in a gradual decline in bone formation and osteoporosis development (Clarke and Khosla 2010; Georgiou et al. 2012). Numerous factors can inhibit osteogenic differentiation and bone formation, with oxidative stress being a notable contributor (Jiang et al. 2020; Isomura et al. 2004; Feng and Tang 2014; Hamada et al. 2007).

Research has demonstrated that aging results in the build‐up of reactive oxygen species (ROS) within cellular structures. The impact of ROS on organisms depends on their levels. Under normal physiological conditions, ROS production is counterbalanced by antioxidant mechanisms, including glutathione peroxidase (GPx), glutathione (GSH), and superoxide dismutase (SOD) (Domazetovic et al. 2017). When ROS levels become excessive or persistent, they overwhelm the antioxidant capabilities of cells or tissues, upsetting the redox equilibrium and causing oxidative harm. Recent investigations have indicated that in the development of aging and age‐related bone disorders, increased ROS generation induces chronic and persistent oxidative stress and diminishes antioxidant capacity, leading to cellular and tissue damage and initiating a cascade of pathological alterations (Steinz et al. 2024). Furthermore, age‐related osteoporosis is closely associated with elevated ROS levels in osteoblasts, and excessive ROS can promote osteoblast apoptosis and reduce bone formation (Bonaccorsi et al. 2018; Becerikli et al. 2017).

The forkhead transcription factor O (FoxO) family comprises transcription factors with a conserved Fork region, including FoxO1, FoxO3, FoxO4, and FoxO6, found in bones, muscles, liver, pancreas, and heart (Eijkelenboom and Burgering 2013). FoxOs regulate energy metabolism, oxidative stress, apoptosis, DNA damage repair, and other biological processes (Santo and Paik 2018; Klotz et al. 2015). FoxO‐mediated resistance to oxidative stress is crucial for maintaining the cell redox balance. Short‐term physiological doses of ROS or stress signals can rapidly trigger defense mechanisms and boost FoxO transcriptional activity.

FoxOs counteract oxidative stress by activating antioxidant systems including GPx, GSH, and SOD, thereby preserving cellular and organ function (Domazetovic et al. 2017; Accili and Arden 2004). FoxO1, the first identified member of the FoxO family, is highly expressed in osteoblasts and regulates bone metabolism (Teixeira et al. 2010). Conditional FoxO1 knockout in osteoblasts leads to bone loss and reduces bone formation in mice, whereas overexpression of FoxO1 increases the levels of osteogenic markers, such as *Runx2* and *Alp*, promoting bone formation (Rached et al. 2010). However, recent research has indicated that excessive ROS, resulting from aging, hinders osteoblast differentiation, leading to osteoporosis and compromised bone healing. Significantly, targeted deletion of FoxO1 in osteoblasts has been found to reduce bone loss in aged mice (Xiong, Zhang, Guo, et al. 2017; Xiong, Zhang, Xin, et al. 2017; Xiong et al. 2022).

MACF1 (microtubule actin crosslinking factor 1), a protein belonging to the spectraplakin family, exhibits reduced expression as humans age. The loss of MACF1 can hasten the development of age‐related osteoporosis, whereas increased MACF1 expression can mitigate this condition (Yin et al. 2021; Zhang et al. 2023; Chen et al. 2022). Furthermore, MACF1 plays a crucial role in the Wnt/β‐catenin signaling pathway, which enhances the differentiation of preosteoblastic cells (Yin et al. 2021; Hu et al. 2017; Qiu et al. 2020; Zhang et al. 2018). Within the canonical Wnt/β‐catenin pathway, MACF1 aids in moving Axin, APC, β‐catenin, and GSK‐3β complexes from the cytoplasm to the cell membrane. This process results in the movement of β‐catenin into the nucleus, where it interacts with LEF/TCF7, its cofactor, to regulate the expression of genes involved in osteogenic differentiation (Chen et al. 2006). MACF1 knockdown decreased Wnt/β‐catenin pathway activity by inhibiting β‐catenin expression and binding to TCF7 (Chen et al. 2006; Ka et al. 2014; Hu et al. 2018). Recent studies have indicated that excessive ROS or chronic stress can redirect β‐catenin from TCF‐ to FoxO‐ mediated transcription, thereby affecting osteoblast function (Essers et al. 2005; Almeida et al. 2007). Nonetheless, it remains uncertain whether the downregulation of MACF1 during age‐related osteoporosis is responsible for the transition of β‐catenin from TCF‐ to FoxO‐ binding.

Given these findings, we utilized MACF1 conditional knockout (MACF1‐cKO) mice as a model for premature aging to investigate how FoxO1 responds to chronic oxidative stress and to elucidate the potential mechanisms by which FoxO1 influences bone metabolism in elderly mice. Herein, we show that MACF1‐cKO mice exhibit chronic and persistent oxidative stress that decreases bone formation, deteriorates microarchitecture, and weakens the mechanical properties of the bone. Moreover, in MACF1 deficient preosteoblastic cells, the expression, nuclear translocation and transcriptional activity of FoxO1 are promoted, and the antioxidant gene expression is upregulated. Mechanistically, chronic oxidative stress induced by MACF1 deficiency enhances the binding of FoxO1 to β‐catenin, which competitively inhibits the binding of β‐catenin to TCF7. This study provides an experimental and theoretical basis for further research on the mechanisms underlying age‐related osteoporosis, revealing that FoxO1 is a potential therapeutic target for the treatment of senile osteoporosis.

## 2 Results

### 2.1 Chronic Oxidative Stress Induced by Excessive ROS Resulted From MACF1 Deficiency Inhibits Osteoblastic Cell Differentiation

**FIGURE 1:**
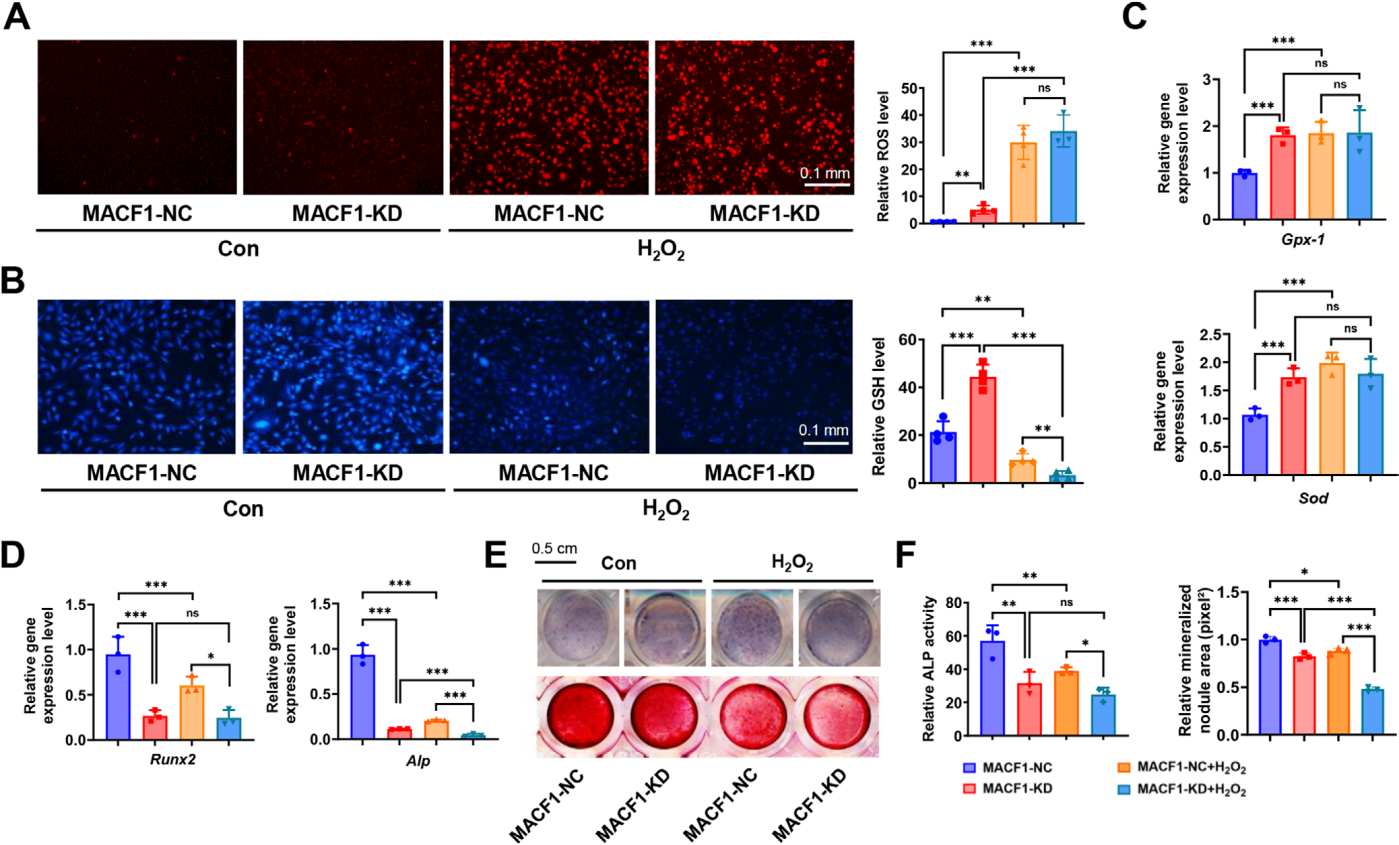
Chronic oxidative stress induced by MACF1 deficiency inhibits osteoblast differentiation. (A) ROS levels and (B) GSH levels in MACF1 knockdown MC3T3‐E1 cells (MACF1‐KD) and control cells (MACF1‐NC) treated with or without 200 μM H_2_O_2_ for 12 h and quantitative analysis. Scale bar, 100 μm. The mRNA expression levels of (C) *SOD* and *GPx‐1* and (D) *Runx2* and *Alp* in MACF1‐NC and MACF1‐KD cells treated with or without H_2_O_2_. (E) Representative ALP staining images (upper) and Alizarin Red S staining images (lower) of MACF1‐NC and MACF1‐KD cells treated with or without H_2_O_2_, Scale bar, 500 μm. (F) Semi‐quantitative analysis of ALP activity and mineralized nodule area. *p < 0.05, **p < 0.01 and ***p < 0.001.

MACF1 knockdown MC3T3‐E1 cell line (MACF1‐KD) and primary cortical bone‐derived mesenchymal stem cells (MSCs) isolated from MACF1 MSCs condition knockout mice (MACF1‐cKO) were treated with H_2_O_2_ to evaluate oxidative stress and ROS levels after MACF1 deficiency. Cell viability was first detected, as shown in Figure S1A,B, the IC50 values of MACF1 deficient cells (MACF1‐KD: 908.1 μM, MACF1‐cKO: 235.8 μM) were significantly decreased compared with control cells (MACF1‐NC: 1048 μM, MACF1‐flox: 406.2 μM). For both MACF1‐NC and MACF1‐KD cells, cell viability remained unchanged when the H_2_O_2_ concentration was < 200 μM. Consequently, 200 μM was chosen as the safe concentration for treating osteoblast cell lines for 48‐h. Similarly, for MACF1‐flox and MACF1‐cKO cells, no significant change in cell viability was observed when the H_2_O_2_ concentration was below 100 μM. So, 100 μM was used for treating primary osteoblasts for 48 h. Following this, osteoblastic cells were treated with a high concentration of H_2_O_2_ 200 μM for 12 h, which can lead to persistent and excessive reactive oxygen species (ROS) similar to aging (Li et al. 2025), and ROS levels were detected. The results showed that ROS levels in MACF1‐KD cells and H_2_O_2_ treated MACF1‐NC cells were significantly higher than those in MACF1‐NC cells. Moreover, ROS levels in H_2_O_2_ treated MACF1‐KD cells were higher than those in untreated MACF1‐KD cells but comparable to those in H_2_O_2_ treated MACF1‐NC cells (Figure 1A). The GSH (Glutathione) levels in MACF1‐KD cells were remarkably higher than those in MACF1‐NC cells. However, the GSH levels in H_2_O_2_ treated MACF1‐KD cells were significantly lower than those in MACF1‐KD cells (Figure 1B). Moreover, the ROS and GSH phenotypes in primary MACF1 knockout MSCs treated with or without H_2_O_2_ were consistent with those of MACF1 knockdown cells (Figure S1C,D). The expression of antioxidant genes (*Sod2* and *Gpx‐1*) in MACF1‐KD cells and H_2_O_2_‐treated MACF1‐NC cells was notably upregulated compared to that in MACF1‐NC cells. However, antioxidant gene expression was not further upregulated in H_2_O_2_‐treated MACF1‐KD cells compared to that in untreated cells (Figure 1C). The alteration in antioxidant gene expression in primary MACF1 knockout MSCs was similar to that in MACF1 knockdown cells (Figure S1E). These results indicate that MACF1 deficiency weakens the oxidation resistance of osteoblasts and reduces their ability to respond to external oxidative stimuli. In addition, the expression of osteogenic differentiation marker genes (*Alp* and *Runx2*) was significantly downregulated in MACF1‐KD cells and H_2_O_2_‐treated MACF1‐NC cells compared with MACF1‐NC cells. However, H_2_O_2_ had a slight effect on the gene expression in MACF1‐KD cells compared to that in untreated MACF1‐KD cells (Figure 1D). Moreover, MACF1‐KD cells and H_2_O_2_ treated MACF1‐NC cells displayed significantly reduced ALP activity and delayed mineralization compared with MACF1‐NC cells. H_2_O_2_ significantly reduced ALP activity and decreased the number of mineralized nodules in MACF1‐KD cells compared to those in the control (Figure 1E,F). Primary MSCs showed a similar mineralization phenotype as MACF1 knockdown MC3T3‐E1 cells (Figure S1F). These data suggest that similar to treatment with high concentrations of H_2_O_2_ for extended periods, the absence of MACF1 in cells results in persistent and high levels of ROS, leading to chronic oxidative stress and inhibiting the differentiation of osteoblastic cells.

### 2.2 NAC Rescued MACF1‐cKO Mice Bone Loss Through Promoting Osteoblastic Cell Differentiation by Enhancing Antioxidant Level and Decreasing ROS Level

**FIGURE 2:**
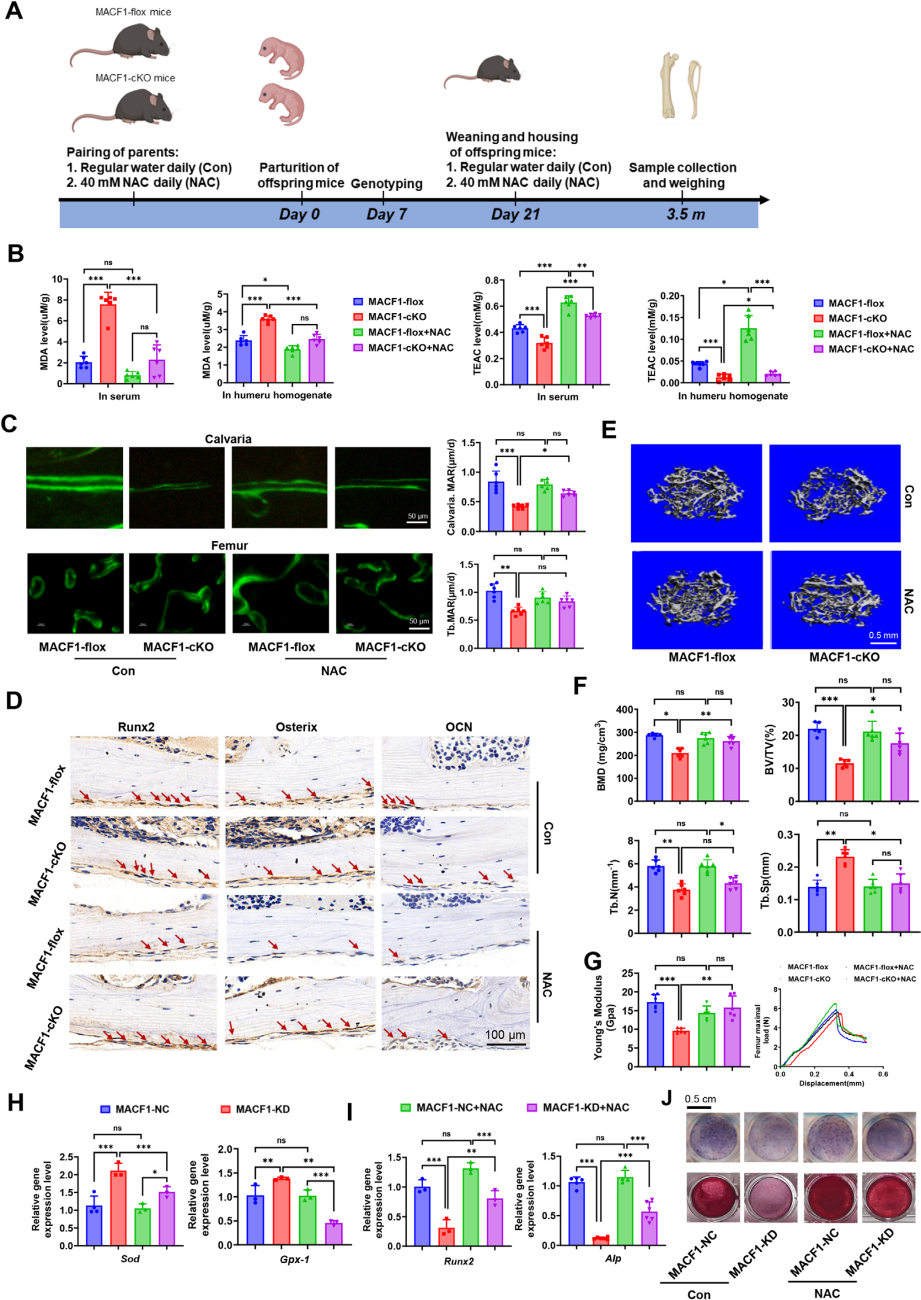
NAC alleviated bone loss in MACF1‐cKO mice and restored MACF1 deficient osteoblastic cells differentiation by decreasing oxidative stress levels. (A) Diagram depicting the administration of NAC treatment in MACF1‐cKO mice from 0 to 3.5 months of age. Analysis of (B) MDA and TEAC levels in serum and humerus homogenates of adult MACF1‐flox and MACF1‐cKO mice treated with or without 40 mM NAC, *n* = 6 in each group. (C) Double calcein labeling images of calvaria (upper) and tibia (lower) showing new bone formation from adult MACF1‐flox and MACF1‐cKO mice, and quantitative analysis. MAR, mineral apposition rate. *n* = 6 in each group. Scale bars, 50 μm. (D) Immunohistochemical staining of Runx2, Osterix, and Ocn in tibial sections from adult MACF1‐flox and MACF1‐cKO mice treated with or without NAC, *n* = 3 in each group; scale bars, 100 μm. (E) Representative trabecular bone 3D μ‐CT images from distal femurs of adult MACF1‐flox and MACF1‐cKO mice. Scale bars, 0.5 mm. (F) Quantitative μ‐CT analysis of BMD, BV/TV, Tb.N, and Tb.Sp in MACF1‐flox and MACF1‐cKO mice (*n* = 6 per group). (G) Young's modulus and femur maximal load of the tibia from adult MACF1‐flox and MACF1‐cKO mice were determined using three‐point bending (*n* = 6 in each group). Expression levels of (H) Sod, Gpx‐1, (I) ALP, and Runx2 in MACF1‐NC and MACF1‐KD cells treated with or without NAC for 48 h. (J) Representative ALP staining images (upper panel) and Alizarin Red S staining images (lower panel) of MACF1‐NC and MACF1‐KD cells treated with or without NAC for 48 h. Scale bar, 500 μm. *p < 0.05, **p < 0.01, ***p < 0.001.

To further corroborate the chronic oxidative stress condition in MACF1‐cKO mice, the survival curve of MACF1‐cKO mice treated with NAC, an antioxidant, was analyzed. The findings indicated that MACF1‐flox mice exhibited no mortality from 0 to 21 months. However, the survival rate of MACF1‐cKO mice gradually decline at 14 months of age and decreased to approximately 50% at the 19 months, while was improved to 100% by NAC treatment (Figure S2A). Subsequently, the impact of NAC treatment on bone tissue performance in MACF1‐cKO mice was investigated (Figure 2A). The results showed that there was no obvious effect of MACF1 knockout or NAC treatment on the weight (Figure S2B) and organ coefficient of mice (Figure S2C). H&E staining of liver, spleen and kidneys showed that NAC did not cause toxicity on liver, spleen and kidneys (Figure S2D). MDA levels in serum and humerus homogenates were significantly upregulated in MACF1‐cKO mice compared with MACF1‐flox mice. Serum MDA levels in both MACF1‐flox and MACF1‐cKO mice fed NAC were dramatically decreased compared to those in normal‐fed mice. TEAC levels in both serum and humerus homogenates of MACF1‐flox and MACF1‐cKO mice fed with NAC were significantly higher than those in normal‐fed mice. Nevertheless, even with NAC supplementation, the levels of MDA and TEAC in MACF1‐cKO mice remained significantly elevated and reduced, respectively, compared with those in NAC‐fed MACF1‐flox controls (Figure 2B). Calcein labeling indicated that bone formation and mineral appositional rates (MAR) in the calvaria and femur tissues were notably reduced in MACF1‐cKO mice compared to MACF1‐flox mice. However, these rates were higher in NAC‐fed MACF1‐cKO mice compared to those fed a normal diet (Figure 2C). Moreover, the levels of the osteoblastic markers Runx2, Osterix, and Ocn decreased in MACF1‐cKO mice compared with MACF1‐flox mice yet increased after feeding with NAC compared with normal‐fed mice (Figure 2D). Micro‐CT analysis of the trabecular architecture showed that MACF1‐cKO mice displayed lower bone mass than MACF1‐flox mice, and NAC‐fed MACF1‐cKO mice displayed higher bone mass than normal‐fed MACF1‐cKO mice (Figure 2E). Detailed analysis of the skeletal phenotype revealed decreased bone mineral density (BMD), bone volume per tissue volume (BV/TV), trabecular number (Tb.N), and increased trabecular space (Tb.Sp) in MACF1‐cKO mice compared to MACF1‐flox mice. NAC‐fed MACF1‐cKO mice displayed a noticeable increase in BMD, BV/TV, and Tb.N and decreased Tb.Sp compared with normal‐fed MACF1‐cKO mice (Figure 2F). Consistently, the femoral strength of adult MACF1‐cKO mice was greatly reduced compared with that of MACF1‐flox mice, while NAC feeding increased it in the MACF1‐cKO mice compared with normal feeding (Figure 2G). Nevertheless, the MAR, bone microstructure, and mechanical properties of NAC‐treated MACF1‐cKO mice were inferior to those of NAC‐treated MACF1‐flox mice. Aged MACF1‐cKO mice fed with NAC (Figure S2E) displayed mechanical properties similar to those of adult MACF1‐cKO mice fed with NAC (Figure S2F). At the cellular level, the expression of antioxidant genes was upregulated in MACF1‐KD cells compared with MACF1‐NC cells and significantly downregulated in MACF1‐KD cells treated with NAC compared with that in untreated cells (Figure 2H). The expression of osteoblastic marker genes was contrary to that of antioxidant genes (Figure 2I). ALP activity and mineralized nodules of MACF1‐KD cells were lower than those of MACF1‐NC cells, and MACF1‐KD cells treated with NAC showed increased ALP activity compared to untreated cells (Figure 2J and Figure S2G). However, the osteogenic differentiation capacity of NAC‐treated MACF1‐KD cells was still lower than that of NAC‐treated MACF1‐NC cells. These findings indicate that NAC partially alleviated the inhibition of osteoblast differentiation and bone formation induced by MACF1 deficiency by decreasing ROS and oxidative stress levels.

### 2.3 FoxO1/β‐Catenin Pathway Activity Was Increased in MACF1 Deficient Osteoblastic Cells

**FIGURE 3:**
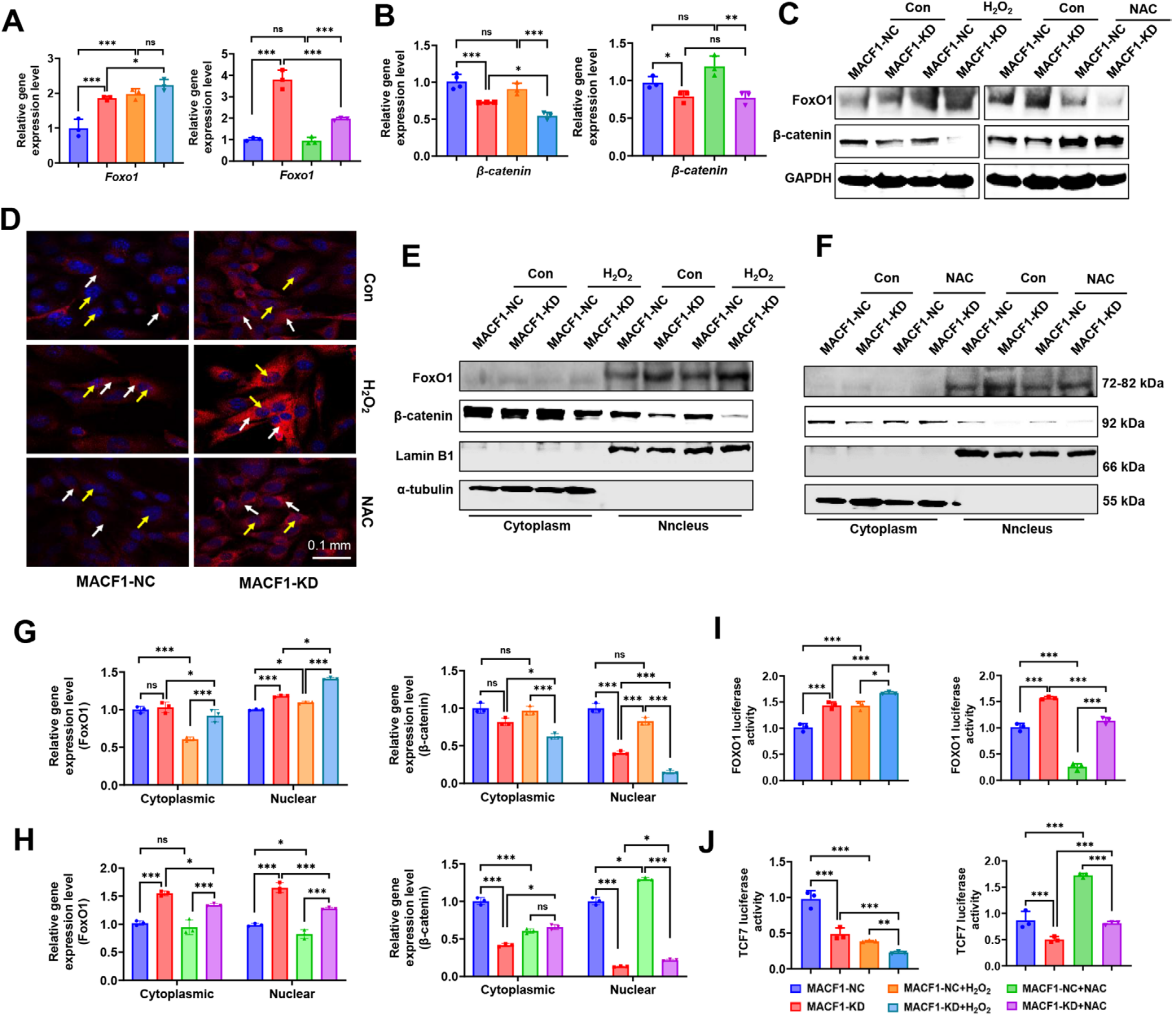
FoxO1/β‐catenin pathway activity increased and TCF7/β‐catenin pathway activity decreased in MACF1 deficient osteoblastic cells. (A) FoxO1 and (B) β‐catenin mRNA expression in MACF1‐NC and MACF1‐KD cells treated with or without H_2_O_2_ or NAC. (C) Immunoblot analysis of β‐catenin and FoxO1 protein levels in MACF1‐NC and MACF1‐KD cells treated with or without H_2_O_2_ (left) and NAC (right). (D) Representative immunofluorescence staining images of FoxO1 in MACF1‐NC and MACF1‐KD cells treated with or without H_2_O_2_. Scale bars, 50 μm; yellow arrow, nuclear FoxO1; white arrow, cytoplasmic FoxO1. (E) β‐catenin and FoxO1 protein levels in the cytoplasm and nucleus of MACF1‐NC and MACF1‐KD cells treated with or without H_2_O_2_ and (G) quantification analysis. (F) β‐catenin and FoxO1 protein levels in the cytoplasm and nucleus of MACF1‐NC and MACF1‐KD cells treated with or without NAC and (H) quantification analysis. (I) Luciferase activity of FoxO1 and (J) TCF7 in MACF1‐NC and MACF1‐KD cells treated with or without H_2_O_2_ and NAC. *p < 0.05, **p < 0.01, ***p < 0.001.

We aimed to elucidate the mechanism by which excessive ROS inhibited osteoblastic cell differentiation induced by MACF1 deficiency, and found that FoxO1 mRNA expression in MACF1‐KD cells was significantly upregulated compared to that in MACF1‐NC cells. While H_2_O_2_ treatment did not further upregulate FoxO1 levels in MACF1‐KD cells compared to untreated cells, NAC treatment downregulated its level compared to untreated cells (Figure 3A). The mRNA expression level of β‐catenin was significantly reduced in MACF1‐KD cells compared to MACF1‐NC cells, and it was further diminished in MACF1‐KD cells treated with H_2_O_2_ compared to that in untreated cells. Conversely, NAC treatment did not significantly affect β‐catenin mRNA levels in either MACF1‐NC or MACF1‐KD cells (Figure 3B). The alteration in mRNA expression levels of TCF7 in NAC‐treated MACF1‐cKO cells was analogous to that of β‐catenin in MACF1 knockdown cells, with the exception of a notable increase observed in MACF1‐cKO cells compared to untreated cells (Figure S3A). The protein expression levels of FoxO1 and β‐catenin were found to be consistent with their corresponding mRNA levels, except for the increased protein level of β‐catenin in NAC‐treated MACF1‐KD cells (Figure 3C, and Figure S3B,C). Notably, regardless of H_2_O_2_ treatment, FoxO1 mRNA and protein levels were elevated in MACF1‐KD cells compared to MACF1‐NC cells. Immunofluorescence staining analysis showed that the fluorescence intensity of FoxO1 was stronger, and nuclear translocation (yellow arrow) was more pronounced in MACF1‐KD cells than in MACF1‐NC cells, and in H_2_O_2_ treated cells compared with that in untreated cells; while in NAC‐treated cells, the fluorescence intensity of FoxO1 and nuclear translocation were declined (Figure 3D and Figure S3D).

Consistently, nuclear‐cytoplasmic separation analysis showed that cytoplasmic FoxO1 levels in MACF1‐KD cells were elevated compared to that in MACF1‐NC cells, and the levels were also increased in both MACF1‐NC and MACF1‐KD cells treated with H_2_O_2_ relative to that in untreated cells. Similarly, nuclear FoxO1 levels in MACF1‐KD cells were elevated compared with those in MACF1‐NC cells and were also increased in both MACF1‐NC and MACF1‐KD cells after H_2_O_2_ treatment compared with those in untreated cells. However, β‐catenin abundance in the nucleus and cytoplasm in both MACF1‐KD cells and H_2_O_2_‐treated cells was opposite to that of FoxO1 (Figure 3E,G). Moreover, NAC treatment recovered FoxO1 and β‐catenin nuclear translocation in MACF1‐KD cells compared with that in untreated cells (Figure 3F,H). The alteration of FoxO1 in MACF1 knockdown cells was confirmed through immunofluorescence staining of FoxO1 in osteoblasts within bone tissue sections from NAC‐treated adult MACF1‐cKO mice (Figure S3E). To further assess the transcriptional activity of FoxO1 and TCF7, a recombinant pNL1.3 reporter plasmid was constructed by inserting a promoter region containing the FoxO1 (or TCF7) recognition sites of the downstream genes of FoxO1 into the pNL1.3 reporter plasmid and then transfected into MC3T3‐E1 cells. The results showed that (Figure 3I), compared with MACF1‐NC cells, the luciferase activity of FoxO1 in MACF1‐KD cells was significantly increased. Furthermore, it also increased in both MACF1‐NC and MACF1‐KD cells treated with H_2_O_2_ and decreased in both cells treated with NAC compared with that in untreated cells, suggesting that NAC reversed the expression and nuclear translocation of FoxO1 promoted by oxidative stress induced by MACF1 deficiency. In contrast, the luciferase activity of TCF7 in MACF1‐KD cells was significantly reduced compared to MACF1‐NC cells, a finding corroborated in primary MSCs derived from MACF1‐cKO mice. Furthermore, the presence of H_2_O_2_ intensified this reduction, whereas NAC effectively mitigated this effect (Figure 3I and Figure S3F). These results demonstrate that FoxO1 responds to excessive ROS and enhances the FoxO1/β‐catenin pathway to mitigate oxidative stress.

### 2.4 Up‐Regulated FoxO1 Seizes β‐Catenin, Impeding Binding of TCF7 to β‐Catenin After MACF1 Deficiency

**FIGURE 4:**
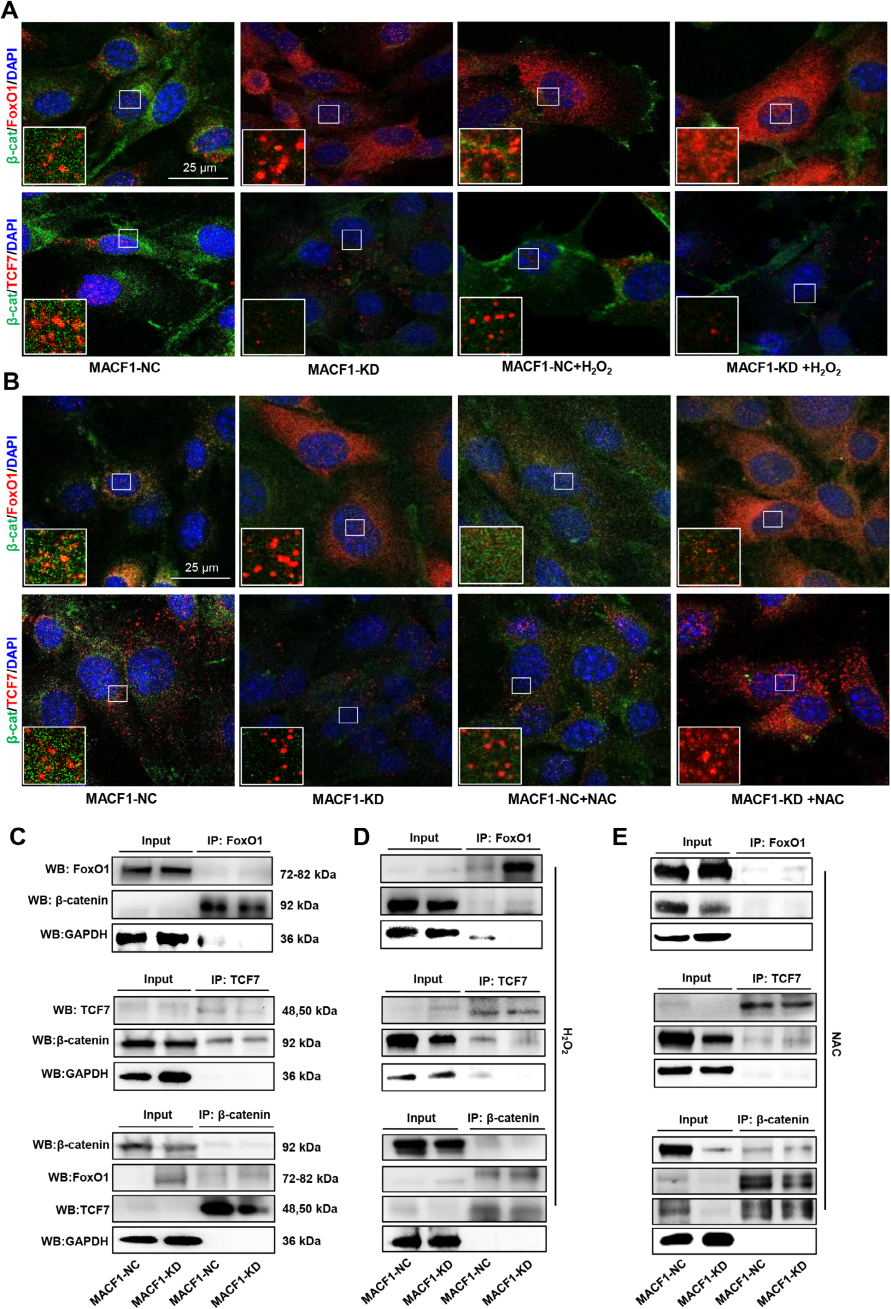
Upregulated FoxO1 binds to β‐catenin, decreasing the binding of TCF7 to β‐catenin in MACF1 knockdown osteoblastic cells. (A, B) Representative immunofluorescence staining images of FoxO1, TCF7, and β‐catenin in MACF1‐NC and MACF1‐KD cells treated with or without H_2_O_2_ or NAC. Scale bars, 25 μm. (C–E) Co‐IP of FoxO1, β‐catenin, and TCF7 in MACF1‐NC and MACF1‐KD cells treated with or without H_2_O_2_ or NAC.

We further evaluated the destination of β‐catenin to FoxO1 or TCF7 in MACF1 deficient osteoblastic cells. Immunofluorescence staining and co‐IP were used to examine the competitive binding of FoxO1 and TCF7 to β‐catenin. The results showed that, compared with MACF1‐NC cells, MACF1‐KD cells exhibited not only reduced fluorescence intensity of β‐catenin but also enhanced intensity of FoxO1. Moreover, FoxO1 was found to colocalize with β‐catenin. For TCF7/β‐catenin binding, compared with MACF1‐NC cells, the fluorescence intensity of TCF7 in MACF1‐KD cells was diminished, and colocalization with β‐catenin was decreased as well. Subsequently, preosteoblastic cells were treated with H_2_O_2_, and colocalization of FoxO1/β‐catenin and TCF7/β‐catenin was detected. The results showed that, compared to non‐ H_2_O_2_ treated cells, in H_2_O_2_ treated cells, the colocalization of FoxO1 with β‐catenin was enhanced although β‐catenin fluorescence intensity was reduced. Concurrently, the fluorescence intensity of TCF7 was further weakened and its colocalization with β‐catenin was further reduced. These results suggest that the knockdown of MACF1 enhances the binding of FoxO1 to β‐catenin, thereby competitively weakening the binding of TCF7 to β‐catenin. Moreover, H_2_O_2_ further significantly intensified the binding of FoxO1 to β‐catenin, thereby weakening the binding of TCF7 to β‐catenin (Figure 4A and Figure S4A). To validate these results, MACF1‐NC and MACF1‐KD cells were treated with NAC, and the colocalization of FoxO1/β‐catenin and TCF7/β‐catenin was detected. The results showed that, compared to non‐NAC‐treated cells, in NAC‐treated cells, the fluorescence intensity of FoxO1 was reduced, its colocalization with β‐catenin was somewhat decreased, and the colocalization of TCF7 with β‐catenin was enhanced along with increased TCF7 fluorescence intensity, although the fluorescence intensity of β‐catenin was not completely restored (Figure 4B and Figure S4B). These results indicated that NAC partially reduced the increased colocalization of FoxO1/β‐catenin and the decreased colocalization of TCF7/β‐catenin caused by MACF1 deficiency. Subsequently Co‐IP experiments were performed using specific antibodies against FoxO1, TCF7, or β‐catenin. The results showed that β‐catenin was present in the FoxO1 antibody precipitate, and its signal intensity was higher in MACF1‐KD cells than in MACF1‐NC cells. In contrast, β‐catenin signal in the TCF7 antibody precipitate was diminished in MACF1‐KD cells compared with that in MACF1‐NC cells. This indicated that in MACF1 deficient preosteoblast cells, the interaction between FoxO1 and β‐catenin was enhanced, whereas the interaction between TCF7 and β‐catenin was weakened (Figure 4C). Compared to H_2_O_2_ treated MACF1‐NC cells, in H_2_O_2_ treated MACF1‐KD cells, the amount of β‐catenin in the FoxO1 antibody precipitate was increased, whereas it was decreased in the TCF7 antibody precipitate, suggesting that MACF1 deficiency enhanced the binding of FoxO1/β‐catenin and competitively weakened TCF7/β‐catenin binding. H_2_O_2_ treatment further strengthened the binding of FoxO1 to β‐catenin, but weakened TCF7/β‐catenin binding (Figure 4D). As expected, NAC partially re‐balanced the binding of FoxO1/β‐catenin and TCF7/β‐catenin in MACF1‐KD cells compared to that in NAC‐treated MACF1‐NC cells (Figure 4G). These results indicate that the upregulation of FoxO1 induced by high levels of ROS deprives β‐catenin from TCF7, thereby competitively suppressing TCF7/β‐catenin pathway. Consequently, the oxidative stress induced by MACF1 deficiency modifies the destination of β‐catenin, leading to a functional switch in pre‐osteogenic cells from “osteogenic differentiation” to “antioxidation.”

## 3 Discussion

This study revealed a critical role of FoxO1 in aging‐related osteoporosis. Persistent and chronic oxidative stress induced by MACF1 deficiency upregulates FoxO1 expression, and inhibits osteoblast differentiation and bone formation. Under oxidative stress conditions, elevated FoxO1 deprives β‐catenin from TCF7, inhibiting the Wnt/β‐catenin pathway and suppressing osteoblast differentiation. These results establish a sequence of molecular events in which FoxO1 negatively regulates osteoblast function under persistent and chronic oxidative stress conditions. More importantly, they revealed the regulatory effect of MACF1 on the FoxO1/β‐catenin pathway, osteoblast function, and mechanisms of aging‐related osteoporosis.

In adult vertebrates, bone remodeling is an ongoing process that involves two consecutive cellular activities. Osteoclasts break down the mineralized bone matrix through resorption, followed by the formation of new bone by osteoblasts (Stegen and Carmeliet 2024). In osteoporosis, this remodeling process is disrupted, resulting in low bone mass due to an imbalance between bone formation and resorption (Rodan and Martin 2000). Starting in their mid‐40s, both men and women experience a decline in bone mass and strength, which accelerates in women during menopause due to reduced estrogen levels (Riggs et al. 2006; Bouxsein et al. 2006). Therefore, osteoporosis is a condition frequently associated with aging.

Oxidative stress results from increased levels of ROS, such as superoxide anions, hydroxyl radicals, and hydrogen peroxide (Cheng et al. 2016). Elevated ROS levels can damage proteins, lipids, and DNA, possibly causing cell death or activating specific signaling pathways (Wang et al. 2021). Physiological stress triggers defense mechanisms that maintain cellular and organismal function. ROS‐induced damage and activation of signaling pathways influence many cellular processes related to longevity in invertebrates and vertebrates (Kozlov et al. 2024; Juan et al. 2021). Research has identified a link between aging, age‐related diseases, and increased oxidative stress, highlighting their roles in development (Barrientos 2012). Osteoporosis is associated with increased oxidative stress in osteoblasts, suggesting its involvement in the pathophysiology of bone loss. An osteoporotic phenotype linked to oxidative damage has been observed in mouse models of premature aging (Rached et al. 2010; Tyner et al. 2002).

MACF1 is a crucial cytoskeletal cross‐linking protein that facilitates the connection between microtubules and actin filaments to regulate cellular functions (Ka et al. 2014; Hu et al. 2016). A previous study reported that in neurons, the phenotypic decay of the microtubule (MT) cytoskeleton precedes other aging phenotypes, such as the decline of axons and synaptic terminals. The loss of function of MT tip‐binding proteins, including MACF1, triggers the decay of MT bundles during aging, whereas the improvement of MT networks can alleviate neuronal aging. This indicates that MACF1 is a key site for cellular damage during aging (Okenve‐Ramos et al. 2024).

In our previous investigation, we observed that the expression of MACF1 in the femoral tissue of elderly patients with osteoporosis exhibited an age‐dependent decline. Furthermore, we identified a positive association between *Macf1* expression and the expression levels of both *Ocn* and *Osterix* (Yin et al. 2021). MACF1 deficiency exacerbated age‐related osteoporosis, while MACF1 overexpression rescued age‐related and postmenopausal osteoporosis by promoting osteoblastic cell differentiation (Zhang et al. 2023; Chen et al. 2022). In this study, we found that MACF1 knockout led to chronic oxidative stress throughout the body of mice at the animal level. Meanwhile, MACF1‐deficient cells exhibited high levels of ROS and low levels of GSH, which could be simulated by treatment with a high concentration of H_2_O_2_ (200 μM) for 12 h, suggesting that MACF1 deficiency induced persistent and excessive ROS production and triggered redox feedback. However, H_2_O_2_ treatment did not further increase ROS levels, but even decreased GSH levels in MACF1‐deficient cells, suggesting that the level of ROS induced by MACF1 deficiency surpassed the capacity of redox balance regulation in osteoblastic cells. Therefore, the use of MACF1‐cKO mice and MACF1 knockdown MC3T3‐E1 cells as an accelerated aging model to reveal the mechanism of age‐related osteoporosis is reasonable.

NAC is widely used to remove ROS (Lei et al. 2012). In our study, NAC pretreatment effectively reduced the redox level, relieved bone loss, improved the microstructure of bone tissue, and enhanced the mechanical properties of bone in MACF1‐cKO mice. At the cellular level, NAC ameliorates the abnormal oxidative stress caused by MACF1 deficiency in osteoblasts. Subsequent ALP activity, RT‐qPCR, and western blotting assays revealed that NAC pre‐treatment rescued the impaired ALP activity to a level similar to that of the WT group, indicating that NAC effectively alleviated the adverse effects on osteogenic differentiation caused by MACF1 deficiency. Nevertheless, the bone formation observed in NAC treated MACF1‐cKO mice, as well as the osteogenic differentiation capacity of NAC treated MACF1‐KD cells, was markedly lower than NAC treated MACF1‐flox mice and MACF1‐NC cells, respectively. This suggests NAC only partially alleviated the impact of MACF1 deficiency on osteoblast differentiation and bone formation.

FoxO1‐mediated antioxidant response is essential for maintaining cellular redox homeostasis under physiological conditions (Chen et al. 2021; Delpoux et al. 2021). In osteoblastic cells, FoxO1 interacts with the transcription factor ATF4 and regulates stress‐dependent pathways of p53 signaling, thereby reducing oxidative stress levels to promote osteoblast differentiation and bone formation (Rached et al. 2010; Ma et al. 2024; Ambrogini et al. 2010). Moreover, FoxO1 activation promotes fracture healing and bone regeneration by reducing ROS in MSCs (Yang et al. 2021). Bone tissue‐specific FoxO1 knockout leads to a decrease in osteoblast number and reduced bone formation capacity (Xiong et al. 2022; Ma et al. 2020). However, a recent study found that FoxO1 had opposing effects on bone formation in young and aging mice (Essers et al. 2005; Almeida et al. 2007). In young mice, knockout of the FoxO1 gene inhibits osteoblastic cell differentiation, leading to a decrease in the number of osteoblasts and a reduction in the bone formation rate, ultimately leading to bone loss and delayed healing of bone defects. In contrast, in aging mice, the lack of FoxO1 alleviated age‐related bone loss and improved the healing of bone defects, suggesting that FoxO1 may have a “biphasic regulatory” effect on bone metabolism. A possible reason for the “biphasic regulatory” effect of FoxO1 may be related to ROS levels. The transcriptional activity of FoxO1 can be enhanced through short‐term stimulation with physiological doses of ROS to upregulate the expression of ROS‐scavenging enzymes and DNA damage repair genes, thereby maintaining the physiological functions of cells and organs (Accili and Arden 2004). However, under oxidative stress conditions induced by excessive ROS, the FoxO1‐mediated antioxidant pathway may inhibit osteogenic differentiation and bone formation. In this study, we found that FoxO1 expression was upregulated by excessive ROS in MACF1‐deficient osteoblastic cells, and H_2_O_2_ treatment further aggravated this change, whereas NAC treatment partially reversed it. Correspondingly, for osteoblastic cell function, H_2_O_2_ treatment exacerbated the inhibition of MACF1 deficiency on osteoblastic cell differentiation, whereas NAC treatment ameliorated this aggravation. These results are consistent with previous reports and suggest that FoxO1 responds to persistent and excessive levels of ROS induced by MACF1 deficiency to inhibit osteoblast differentiation.

The Wnt/β‐catenin signaling pathway is crucial for the regulation of osteoblast differentiation and bone formation (Hu et al. 2024). Its diminished activity significantly contributes to the development of age‐related osteoporosis (Oh et al. 2023). MACF1 has been identified as a vital component of this pathway, which regulates Wnt/β‐catenin pathway activity by promoting stability and nuclear translocation (Chen et al. 2006). The activation of the Wnt/β‐catenin signaling pathway is contingent upon the stability and nuclear translocation of β‐catenin, both of which are modulated by GSK3β. Active GSK3β phosphorylates β‐catenin at Thr41, Ser37, and Ser33, causing the ubiquitination and subsequent degradation of β‐catenin, thereby inhibiting its nuclear translocation and suppressing the Wnt/β‐catenin signaling pathway. The absence of MACF1 triggers this core degradation of β‐catenin mediated by GSK3β (Chen et al. 2006). Our previous studies have demonstrated that the knockout of MACF1 results in reduced activity of the Wnt/β‐catenin pathway by triggering the core degradation of β‐catenin, thereby impeding osteoblast differentiation and bone formation. However, the application of the Wnt/β‐catenin pathway activator lithium chloride, as well as the overexpression of MACF1, can mitigate the inhibitory effects associated with MACF1 deficiency (Hu et al. 2018; Yin et al. 2018).

Research has found that in aging mouse preosteoblasts, high levels of ROS promote the transfer of β‐catenin from TCF4 to FoxO1‐mediated transcription, thereby inhibiting the Wnt/β‐catenin signaling pathway and leading to decreased osteogenic activity. Conversely, the deficiency of FoxO1 indirectly promoted the binding between β‐catenin and TCF4, which in turn activated the Wnt/β‐catenin signaling pathway, thereby alleviating age‐related bone loss and improving the healing of bone defects (Xiong et al. 2022). In addition, MACF1 deficiency enhanced the binding of FoxO1 to β‐catenin and activated the FoxO1/β‐catenin pathway. MACF1 deficiency inhibited the binding of TCF7 to β‐catenin and decreased the activity of the Wnt/β‐catenin pathway, ultimately suppressing osteogenic differentiation of MC3T3‐E1 cells. NAC rescued MACF1‐KD cell differentiation and MACF1‐cKO mouse bone formation by reversing the expression and nuclear translocation of FoxO1 and restoring the binding of TCF7 to β‐catenin. These results indicate that MACF1 deficiency leads to sustained high levels of ROS in mice, causing the transfer of β‐catenin from TCF7 to FoxO1‐mediated transcription and indirectly inhibiting Wnt/β‐catenin pathway activity.

Our previous research shows that MACF1‐KD cells exhibit reduced osteogenic differentiation ability, accompanied by downregulation of TCF7 and β‐catenin, which leads to suppression of the Wnt/β‐catenin signaling pathway (Hu et al. 2018). In this study, we found that under chronic oxidative stress conditions induced by MACF1 deficiency, both the expression and nuclear translocation of FoxO1 were promoted, competitively binding to β‐catenin and further inhibiting the Wnt/β‐catenin signaling pathway, disrupting the equilibrium between the two pathways. This indicates that the synergistic effect of downregulation of the Wnt/β‐catenin pathway and upregulation of the FoxO1/β‐catenin pathway induced by MACF1 deficiency contributes to the suppression of pre‐osteoblast differentiation. Subsequent NAC treatment suppressed the FoxO1/β‐catenin pathway while enhancing the Wnt/β‐catenin pathway due to rebalancing the pathways. While, it is important to note that NAC treatment only partially restored the Wnt/β‐catenin pathway, which may result from the degradation of β‐catenin induced by MACF1 deficiency through core degradation mechanism. Furthermore, the stability and nuclear translocation of β‐catenin are inhibited by reduced Akt activity under chronic oxidative stress conditions, through activating GSK3β and directly phosphorylating β‐catenin at Ser552 (Beurel et al. 2015; Gotz et al. 2025; He et al. 2023; Leyane et al. 2022). In this study, we observed that H_2_O_2_ treatment also slightly caused the reduction of both total and nuclear β‐catenin levels, probably due to the influence of ROS on Akt activity. Following NAC treatment, β‐catenin levels increased a bit, which means that NAC enhances the cellular redox state by scavenging intracellular ROS, thereby inhibiting β‐catenin degradation induced by oxidative stress through multiple mechanisms (Chao et al. 2024; Wu et al. 2022). However, NAC is ineffective against β‐catenin degradation caused by MACF1 deficiency, because the GSK3β pathway is the most critical for controlling β‐catenin degradation.

## 4 Conclusion

**FIGURE 5:**
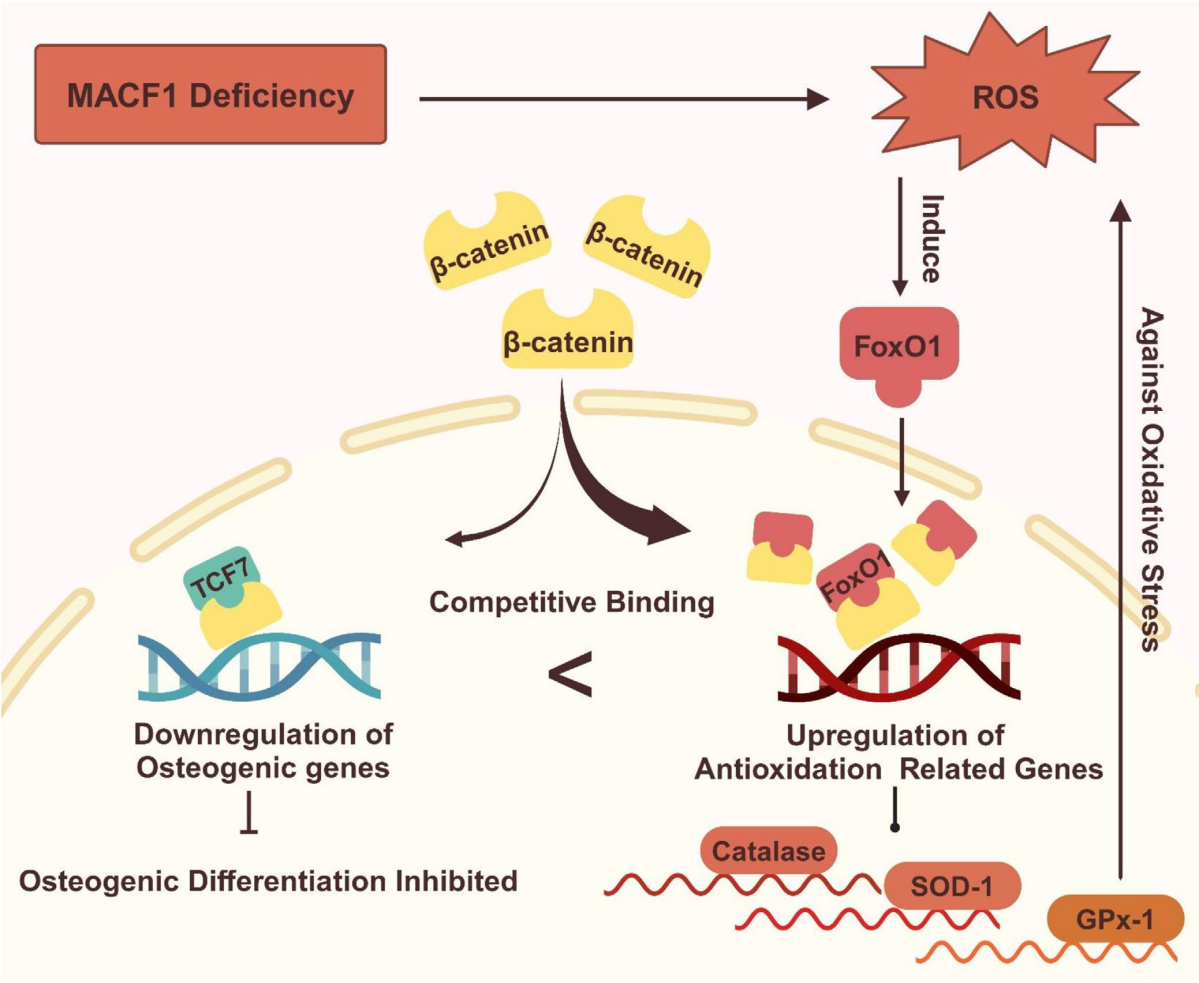
The scheme of FoxO1 responses to chronic oxidative stress caused by MACF1 deficiency to inhibit osteogenic differentiation. Chronic oxidative stress induced by MACF1 deficiency upregulates FoxO1 expression, thus promoting its nuclear translocation and depriving β‐catenin from TCF7, thereby inhibiting TCF7 transcriptional activity and osteogenic differentiation.

In summary, using MACF1‐cKO mice and MACF1‐KD cells as a premature aging model, this study found that FoxO1 responded to persistent and high levels of ROS thus inhibiting osteoblastic cell differentiation and bone formation in MACF1‐cKO mice by depriving β‐catenin from TCF7 (Figure 5). This study reveals a novel regulation of MACF1 to FoxO1 function in aging‐related bone loss, which provides a new perspective for alleviating bone loss in aged patients.

## 5 Method

### 5.1 Experimental Animals and Breeding

MACF1‐flox mice were generously provided by Professor Xiaoyang Wu from the University of Chicago. Prx1‐Cre mice were donated by Professor Ge Zhang from Hong Kong Baptist University. Wild‐type C57BL/6 mice were purchased from the Experimental Animal Center of the Air Force Medical University. Breeding of the MACF1‐cKO mouse model was performed as previously described (Hu et al. 2018; Yin et al. 2018). All mice were maintained and bred in a specific pathogen‐free facility, and animal experiments were conducted in accordance with the institutional guidelines and approval from the Committee of Animal Ethics and Experimental Safety.

In the NAC administration of mice, both parent MACF1‐flox and MACF1‐cKO mice were co‐housed in two separate groups: a control group receiving regular water daily and an experimental group receiving water supplemented with 40 mM NAC daily. After weaning and separation at 21 days post‐birth, the offspring were individually housed and continued to receive NAC treatment until they reached 3.5 months of age. Seven and 2 days before euthanasia at 3.5 months of age, the offspring mice were intraperitoneally injected with calcein. At 3.5 months of age, the offspring mice were euthanized and categorized into four groups based on drinking water and genotype: MACF1‐flox, MACF1‐flox+NAC, MACF1‐cKO, and MACF1‐cKO + NAC groups. Mouse tissues were collected, stored, and preserved for subsequent analysis. For the survival curve analysis, the offspring were housed individually and continued to receive NAC treatment until they reached 21 months of age. Mortality rates were assessed monthly.

Additionally, 20‐month‐old MACF1‐flox and MACF1‐cKO mice were administered 40 mM NAC until they reached 24 months of age, following the same group allocation.

### 5.2 Cell Culture and Reagents

The stably transfected mouse preosteoblastic cell lines, designated MACF1‐NC and MACF1‐KD, were cultured in α‐MEM culture medium supplemented with 2.2 g/mL sodium bicarbonate, 10% fetal bovine serum, 100 μg/mL streptomycin sulfate, 100 units/mL penicillin, 10 mM/L glutamine, 50 μg/mL vitamin C, and 10 mM β‐glycerophosphate sodium. The cultures were maintained in a humidified incubator at 37°C and 5% CO_2_.

Primary cortical bone‐derived mesenchymal stem cells (MSCs) were isolated by extracting the humerus, femur, and tibia of euthanized mice using sterile 100 mm glass dishes. Subsequently, the extracted cells were transferred to a 60 mm sterile dish containing a buffer solution for further pruning and digestion. The experiment utilized cells from the 3rd to 8th generation.

### 5.3 MTT Assay

The concentration of the cell suspension was adjusted to 3 × 10^5^ cells/mL and 200 μL was inoculated into a 96‐well plate. Cells were cultured for 48 h in a humidified incubator. Following this incubation period, the medium was replaced with fresh complete medium containing varying concentrations of hydrogen peroxide (H_2_O_2_). After another 48‐h culture period, 200 μL of complete medium and 20 μL of MTT were added to each well and incubated for a further 4 h. Subsequently, the supernatant was removed and 150 μL of dimethyl sulfoxide was added to each well. The optical density of each well was measured at a wavelength of 490 nm after shaking for 10 min.

### 5.4 Techniques for Labeling With Fluorescent Dyes

For the detection of reactive oxygen species (ROS), 8 × 10^4^ cells per well were inoculated into 48‐well plates. After a 24‐h culture period, the medium was removed. The experimental group was treated with complete medium supplemented with 200 μM H_2_O_2_, while the control group received complete medium without supplementation. After 1.5 h, the supernatant was discarded. Subsequently, complete medium containing 5 μM CellROX Green or DHE fluorescent dye was added and the cells were incubated in a cell incubator at 37°C with 5% CO_2_ for 30 min. The cells were washed with PBS and observed under an inverted fluorescence microscope. For the detection of glutathione (GSH), 2 × 10^4^ cells were inoculated into each well of a 48‐well plate, employing the same culture methodology used for ROS detection. The GSH fluorescent dye, monochlorobimane (100 μM), was added to the complete medium. Following a 3‐h incubation period, the cells were examined using an inverted fluorescence microscope.

### 5.5 Alkaline Phosphatase Staining

Following a three‐day period of osteogenic differentiation after treatment with 100/200 μM H_2_O_2_ for 48 h or 2 mM NAC for 24 h, the cells were rinsed with PBS, and a 4% paraformaldehyde (PFA) solution was applied to each well for fixation. The alkaline phosphatase staining solution required a ratio of 1:2:300 for the BCIP, NBT, and alkaline phosphatase chromogenic buffers. The cells were then stained in the dark. Once blue‐purple crystals became visible, the staining solution was removed and the cells were washed and dried under running tap water prior to scanning with a scanner.

### 5.6 Alizarin Red S Staining

Following a 14‐day period of osteogenic differentiation after treatment with 100/200 μM H_2_O_2_ for 48 h or 2 mM NAC for 24 h, cells were rinsed with PBS. Subsequently, the cells were fixed with a 4% PFA solution, which was then allowed to stain at room temperature for 10–20 min. After the staining process, the dye was removed and the sample was rinsed gently with tap water three to five times. The samples were then dried at room temperature and scanned using a scanner.

### 5.7 RNA Extraction and qPCR Analysis

Total RNA was extracted from the cell samples using the Total RNA Extraction Kit II (OMEGA Bio‐tek). Next, reverse transcription of RNA was performed using the PrimeScript RT Reagent Kit (Perfect Real Time) to synthesize complementary DNA (cDNA). Detection was performed using a C‐1000 Thermal Cycler. The reverse transcription polymerase chain reaction (RT‐PCR) protocol included the following steps: pre‐denaturation at 95°C for 30 s, denaturation at 95°C for 10 s, annealing at 50°C for 20 s, and extension at 72°C for 5 s. A total of 45 amplification cycles were performed with readings taken at 80°C for 2 s. The amplification results were analyzed using the 2^−∆∆CT^ method.

### 5.8 Hematoxylin and Eosin (H&E) Staining

After the paraffin sections were dewaxed and rehydrated, they were stained with a hematoxylin solution. Subsequently, the sections were rinsed with tap water, differentiated using a differentiation solution, and rinsed again with tap water before returning to a blue hue with a blue solution. The specimens were then sequentially dehydrated using an alcohol gradient and stained with eosin dye solution. Following dehydration, samples were mounted with neutral gum. The cells were observed under an inverted fluorescence microscope.

### 5.9 Malondialdehyde (MDA) Assay

After preparing a 0.37% TBA storage solution and working solution for malondialdehyde (MDA) detection, the standard substance was diluted to concentrations of 1, 2, 5, 10, 20, and 50 μM. Subsequently, 0.1 mL of the standard substance at the specified concentrations was added to construct the standard curve, along with 0.1 mL of the sample. Following this, 0.2 mL of the MDA detection working solution was incorporated into the mixture. The resulting solution was heated at 100°C for 15 min, cooled to room temperature, and centrifuged. From each tube, 200 μL of the supernatant was extracted, transferred to a 96‐well plate, and the absorbance at 532 nm was measured using a spectrophotometer.

### 5.10 Assessment of Total Antioxidant Capacity (T‐AOC)

A 10 mM Trolox standard solution was diluted with PBS to achieve final concentrations of 0.15, 0.3, 0.6, 0.9, 1.2, and 1.5 mM. The H_2_O_2_ solution was diluted 1000‐fold using ddH₂O water and the peroxidase was diluted 10‐fold with the detection buffer. The peroxidase working solution (20 μL) was added to each well of a 96‐well plate. Subsequently, 10 μL of PBS, 10 μL of Trolox standard solution at varying concentrations, and 10 μL of each sample were added to the designated blank control, standard curve detection, and sample detection wells, respectively. The contents of each well were gently mixed. Subsequently, 170 μL of ABTS working solution was added to each well, and the mixture was gently agitated. After incubation for 6 min at room temperature, absorbance was measured at 414 nm using a spectrophotometer.

### 5.11 Immunohistochemistry (IHC)

Paraffin sections were dewaxed and rehydrated. Following natural cooling, the sections were transferred to PBS and washed three times using a decolorizing shaker. The slices were then immersed in a 3% hydrogen peroxide solution, incubated for 25 min at room temperature, and protected from light. Subsequently, the slices were transferred back to PBS and washed three times with a decolorizing shaker. A 3% solution of bovine serum albumin (BSA) was applied to the histochemical ring to ensure uniform coverage of the tissue section, and the preparation was incubated at room temperature for 30 min. After the addition of the primary antibody to the slices, the slices were placed flat within a humidified chamber and incubated overnight at 4°C. The slices were then washed three times using a decolorizing shaker, after which the slices were treated with the secondary antibody at room temperature for 50 min. Subsequently, a freshly prepared 3,3′‐diaminobenzidine (DAB) color‐developing solution was added. The duration of color development was monitored under a microscope and the sections were rinsed with tap water to halt the color development process.

### 5.12 Calcein Fluorescence Imaging

The initial segment of the fresh skull tissue was fixed in 4% PFA for 24 h. The tissue was rinsed with running water for 1 h and subsequently immersed in 50% sucrose solution at 4°C for an additional 24 h. The tissue was then rapidly frozen in liquid nitrogen for 2 min, sliced to a thickness of 5 μm, and allowed to air dry at room temperature for 1–2 h. Non‐decalcified hard tissue sections of the skull and femur were prepared and embedded in plastic. Following fixation with 4% PFA for 48 h, the bone tissue was dehydrated using a graded ethanol series. Subsequently, tissues were immersed in different plastic embedding solutions and transferred to an embedding container. An appropriate volume of embedding solution was then added and the bone tissue was subjected to vacuum for 5 h, followed by polymerization in a water bath maintained at 37°C. Slices of 10 μm thickness were obtained and baked overnight at 60°C. The resulting sections were examined and photographed under an inverted fluorescence microscope. The distance between the two lines of bone formation within the skull tissue, as depicted in the image, was measured using ImageJ software (NIH, Bethesda, MD, USA), and the bone formation rate was subsequently analyzed (Ushiku et al. 2010).

### 5.13 A Micro‐Computed Tomography (Micro‐CT) Scan

Femoral tissues from each group were fixed in 4% PFA for 48 h. A GE eXplore Locus micro‐CT system was used for the scanning. Three‐dimensional reconstruction of the bone trabecular region, starting 1 mm distal to the growth plate at the distal end of the femur, was performed, and the following microstructural parameters were analyzed: bone mineral density (BMD), bone volume to tissue volume ratio (BV/TV), trabecular number (Tb.N) and trabecular spacing (Tb.Sp).

### 5.14 Three‐Point Bending Test

The soft tissue surrounding the tibia was surgically excised. The diameters at the upper, middle, and lower points of the tibia were measured using Vernier calipers and were recorded as d1, d2, and d3, respectively. The stiffness and Young's modulus of the tibia were analyzed to establish a linear relationship between the force, deformation, and displacement of the tibial tissue.

### 5.15 Western Blotting

The concentration of the protein samples was determined using a BCA protein quantification kit. Protein samples were combined with 5× SDS‐PAGE loading buffer, heated to 105°C for 10 min, and then rapidly transferred to ice to cool for 2 min. The protein loading per well was standardized to 20 μg, according to the polyacrylamide gel formula. SDS‐PAGE was performed until the smallest band on the protein marker ladder reached the gel bottom. Transfer sandwiches were prepared using a polyvinylidene fluoride (PVDF) membrane, gel, and filter paper that had been immersed in methanol. The sandwiches were subjected to wet transfer at 100 V under constant pressure for 90 min. Following TBST cleaning of the PVDF membrane, a 5% solution of skim milk powder was used to block and transfer the membrane for 60 min. The membrane was washed with TBST three times for 10 min each time, incubated with the primary antibody overnight at 4°C, and washed with TBST three times for 10 min each time. The HRP‐labeled secondary antibody was incubated at room temperature for 1 h and washed three times with TBST for 10 min each time. The luminescent solution was prepared and imaged using a chemiluminescence instrument.

### 5.16 Immunofluorescence Staining

For cell immunofluorescence staining, the cells were seeded onto coverslips in 6‐well plates at a density of 1 × 10^5^ cells/ml and incubated for 12 h. Complete medium was added to the control group, whereas the experimental group was treated with either 200 μM H_2_O_2_ for 12 h or 2 mM NAC for 24 h. Cells were then fixed with 4% PFA for 15 min and subsequently washed three times with Tris‐buffered saline (TBS), with each wash lasting 5 min. The samples were then incubated in TBS containing 0.5% Triton X‐100 (TX) for 10 min, followed by three additional washes. The samples were then blocked with 5% FBS at room temperature for 10 min. The primary antibody against FoxO1 (1:100), TCF7 (1:100) or β‐catenin (1:100) was applied to each coverslip and incubated at 4°C overnight. After incubation, the samples were washed three times. Subsequently, Alexa Fluor 488/Cy3‐conjugated secondary antibody was added to each coverslip and incubated at room temperature for 1 h. The samples were washed three times and stained with DAPI for 10 min. After three additional washes, the coverslips were sealed and the samples were examined using a laser confocal microscope.

For bone sections, paraffin‐embedded samples were treated with xylene twice for 15 min. Then, they were treated with anhydrous ethanol twice for 5 min, followed by 85% and 75% ethanol for 5 min to remove paraffin and rehydrate. The sections were rinsed with distilled water and placed in a pH 8.0 EDTA buffer to retrieve antigens. Antigen retrieval was done using a microwave on medium heat for 8 min, then turned off for 8 min, and then on medium‐low heat for 7 min. After cooling, the sections were washed three times with PBS for 5 min each. The tissue area was marked with a pen, and endogenous peroxidase activity was blocked with 3% H_2_O_2_ at room temperature in the dark for 25 min, followed by 3 times PBS washes of 5 min. Sections were blocked with 3% BSA for 30 min. After blocking, the FoxO1 primary antibody (1:100) was applied andincubated overnight at 4°C in a humidified chamber. After 3 times PBS washes of 5 min, an HRP‐labeled secondary antibody was added and incubated for 50 min. The sections were washed with PBS 3 times for 5 min, treated with Cy3‐TSA at room temperature in the dark for 10 min, and washed with TBST 3 times for 5 min. The sections were then placed in EDTA buffer and microwave‐based antibody stripping, followed by cooling and PBS washing. Nuclei were stained with DAPI at room temperature in the dark for 10 min, washed with PBS 3 times for 5 min, treated with an autofluorescence quenching reagent for 5 min, and rinsed with running water for 10 min. This was followed by 3 times PBS washes of 5 min, and mounting with an anti‐fluorescence quenching medium. Finally, the sections were scanned at wavelengths of 330–380/420 nm (DAPI, blue light) and 510–560/590 nm (Cy3, red light).

### 5.17 The Luciferase Reporter Gene Assay

Cells were inoculated into 6‐well plates and cultured for 12 h. The FHRE luciferase reporter gene plasmid and the pRL‐TK luciferase reporter control plasmid were co‐transfected using the Engreen Entranster H4000 transfection reagent in complete culture medium. Following a 24‐h transfection period, the control group was transitioned to differentiation medium, while the experimental group was transitioned to differentiation medium supplemented with 0.2 mM H_2_O_2_ or 2 mM NAC. Both groups were cultured for 48 h. The cells were then washed three times with PBS. Firefly luciferase activity was measured using a dual‐luciferase assay kit and quantified on a BioTek Synergy microplate reader.

### 5.18 Co‐Immunoprecipitation (CO‐IP)

The cells were harvested and washed twice with pre‐cooled PBS. An appropriate volume of cell lysate supplemented with protease inhibitors was added. Cells were lysed on ice for 30 min, and the lysate was cleared by centrifugation at 10,000–14,000 g for 3–5 min at 4°C. Protein A + G magnetic beads were subjected to magnetic separation and the supernatant was discarded. Subsequently, 500 μL antibody working solution was added. Beads were incubated in a rotating mixer at room temperature for 1 h. Protein A + G magnetic beads were added at a ratio of 20 μL of magnetic bead suspension to 500 μL of the protein sample. The mixture was placed in a rotary mixer and incubated overnight at 4°C. The sample was then placed on a magnetic rack for 10 s to allow for separation, which facilitated the removal of the supernatant. The washing process with the lysis buffer was repeated three times. Finally, 100 μL of SDS‐PAGE Sample Loading Buffer was added to 20 μL of the original volume of magnetic beads, and the mixture was heated at 95°C for 5 min.

### 5.19 Data Analysis

GraphPad Prism 6 software was used for data analysis and graphical representation of results. Initially, a distribution test was performed using the Shapiro–Wilk normality test, which indicated that all data in this study exhibited a normal distribution. Student's *t*‐test was used to analyze the data from the two experimental groups. For experiments involving two or more groups, homogeneity of variance was evaluated using the Brown‐Forsythe test. Subsequently, data were analyzed using one‐way ANOVA, followed by Tukey's multiple comparison test. All data are derived from a minimum of three independent experiments and are presented as mean ± standard deviation (SD). A *p*‐value less than 0.05 was considered indicative of a statistically significant difference.

## Author Contributions

**Peihong Su:** writing‐original draft preparation, methodology, investigation, performing experiments, visualization. **Xiaoli Ma:** performing experiments, investigation. **Chong Yin:** study design, data analysis. **Ruilin Shi:** organize the experimental data and create a mechanism diagram. **Siyu Chen:** organize the experimental data and finalize the drawing notes. **Lihuizi Yang:** manuscript writing and data analysis. **Meng Qu :** data analysis, visualization. **Xinyao Jia:** revised the manuscript. **Qi Yu:** data analysis, visualization. **Hui Li:** investigation, project administration, pathological diagnosis. **Airong Qian:** investigation, project administration, pathological diagnosis. **Ye Tian:** project administration, organization of the studies, supervised the project and correction of the manuscript. All data were generated in‐house, and no paper mill was used. All authors agree to be accountable for all aspects of the research to ensure its integrity and accuracy.

## Funding

This work was supported by the Natural Science Foundation of China (81801871, 32371296, 32371371, and 82204543), the Natural Science Foundation of Guangdong Province (2024A1515013078), the Shaanxi Province Natural Science Foundation (2022JQ‐822), and the Key Scientific Research Project of Education Department of Shaanxi Province (22JS032).

## Conflicts of Interest

The authors declare no conflicts of interest.

## Supporting information


**Figure S1:** MACF1 deficiency decreases the viability of osteoblasts treated with H_2_O_2_ and induces oxidative stress in primary cortical bone‐derived mesenchymal stem cells (MSCs). (A) Viability of MC3T3‐E1 cells treated with 0, 10, 50, 100, 200, 400, 600, 800, 1000 or 1200 μM H_2_O_2_ for 48 h. (B) Primary MSCs treated with 0, 10, 20, 50, 100, 200, 300, 400 or 500 μM H_2_O_2_ for 48 h. MACF1‐flox and MACF1‐cKO MSCs were isolated from the bone tissue of MACF1‐flox mice and MACF1 MSCs conditional knockout mice (MACF1‐cKO). (C) ROS and (D) GSH levels in MACF1‐flox and MACF1‐cKO cells treated with or without 200 μM H_2_O_2_ for 12 h and quantitative analysis. Scale bars, 100 μm. (D) Sod and GPx‐1 mRNA expression in MACF1‐flox and MACF1‐cKO cells treated with or without H_2_O_2_. (F) Representative Alizarin red S staining images and quantification analysis of MACF1‐flox and MACF1‐cKO cells treated with or without H_2_O_2_ and quantification analysis. Scale bars, 500 μm. *p < 0.05, **p < 0.01, ***p < 0.001.
**Figure S2:** NAC had no toxic effects on MACF1‐cKO mice and enhanced the mechanical properties of the bone tissue in aged MACF1‐cKO mice. (A) Survival curve of MACF1‐cKO mice treated with or without NAC, *n* = 10 in each group. (B) Body weight and (C) organ coefficients of MACF1‐flox and MACF1‐cKO mice treated with or without NAC, *n* = 6 in each group. (D) Representative H&E staining images of the spleen, liver, and kidney of MACF1‐flox and MACF1‐cKO mice treated with or without NAC, *n* = 3 in each group; Scale bars, 100 μm. (E) Diagram depicting the administration of NAC treatment in MACF1‐cKO mice from 20 m to 24 m of age. (F) Femur maximal load, Young's modulus, and stiffness of the tibia from aging MACF1‐flox and MACF1‐cKO mice determined using three‐point bending, *n* = 6 in each group. (G) Quantification analysis of ALP activity and area of mineralized nodule. *p < 0.05, **p < 0.01, ***p < 0.001.
**Figure S3:** Quantitative analysis of the expression levels of FoxO1 and β‐catenin, as well as the average intensity of FoxO1. (A) The mRNA level of TCF7 in primary MSCs isolated form MACF1‐cKO mice was detected following treatment with or without NAC. (B) The levels of FoxO1 and β‐catenin in MACF1‐NC and MACF1‐KD cells were assessed following treatment with or without H_2_O_2_/(C) NAC. (D) The average intensity of FoxO1 was measured in MACF1‐NC and MACF1‐KD cells subjected to treatment with or without H_2_O_2_/NAC. (E) Representative immunofluorescence staining images of FoxO1 in bone tissue of MACF1‐cKO mice treated with or without NAC. yellow arrow, FoxO1; C, cortical bone, E, endosteum. Scare bar: 20 μm. (F) Luciferase activity of TCF7 in primary MSCs isolated form bone tissue of MACF1‐cKO mice. **p < 0.01, ***p < 0.001.
**Figure S4:** Quantitative analysis of colocalization. (A) Colocalization of TCF7/β‐catenin and FoxO1/β‐catenin in MACF1‐NC and MACF1‐KD cells treated with or without H_2_O_2_/(B) NAC. *p < 0.05, **p < 0.01, ***p < 0.001.

## Data Availability

Data sharing not applicable to this article as no datasets were generated or analysed during the current study.
