# Supplementary material for: FoxO1 Responses to Chronic Oxidative Stress to Participate in Age‐Related Osteoporosis by Depriving β‐Catenin From TCF7
Source: Aging Cell. 2025 Dec 9;25(1):e70306. doi: 10.1111/acel.70306 (PMC12740996; doi:10.1111/acel.70306)
Supplement: Supplementary file 1 — Figure S1: MACF1 deficiency decreases the viability of osteoblasts treated with H2O2 and induces oxidative stress in primary cortical bone‐derived mesenchymal stem cells (MSCs). (A) Viability of MC3T3‐E1 cells treated with 0, 10, 50, 100, 200, 400, 600, 800, 1000 or 1200 μM H2O2 for 48 h. (B) Primary MSCs treated with 0, 10, 20, 50, 100, 200, 300, 400 or 500 μM H2O2 for 48 h. MACF1‐flox and MACF1‐cKO MSCs were isolated from the bone tissue of MACF1‐flox mice and MACF1 MSCs conditional knockout mice (MACF1‐cKO). (C) ROS and (D) GSH levels in MACF1‐flox and MACF1‐cKO cells treated with or without 200 μM H2O2 for 12 h and quantitative analysis. Scale bars, 100 μm. (D) Sod and GPx‐1 mRNA expression in MACF1‐flox and MACF1‐cKO cells treated with or without H2O2. (F) Representative Alizarin red S staining images and quantification analysis of MACF1‐flox and MACF1‐cKO cells treated with or without H2O2 and quantification analysis. Scale bars, 500 μm. *p < 0.05, **p < 0.01, ***p < 0.001. Figure S2: NAC had no toxic effects on MACF1‐cKO mice and enhanced the mechanical properties of the bone tissue in aged MACF1‐cKO mice. (A) Survival curve of MACF1‐cKO mice treated with or without NAC, n = 10 in each group. (B) Body weight and (C) organ coefficients of MACF1‐flox and MACF1‐cKO mice treated with or without NAC, n = 6 in each group. (D) Representative H&E staining images of the spleen, liver, and kidney of MACF1‐flox and MACF1‐cKO mice treated with or without NAC, n = 3 in each group; Scale bars, 100 μm. (E) Diagram depicting the administration of NAC treatment in MACF1‐cKO mice from 20 m to 24 m of age. (F) Femur maximal load, Young's modulus, and stiffness of the tibia from aging MACF1‐flox and MACF1‐cKO mice determined using three‐point bending, n = 6 in each group. (G) Quantification analysis of ALP activity and area of mineralized nodule. *p < 0.05, **p < 0.01, ***p < 0.001. Figure S3: Quantitative analysis of the expression levels of FoxO1 and β‐catenin, as well as the [file ACEL-25-e70306-s001.zip › acel70306-sup-0001-Figures/acel70306-sup-0001-Supinfo1@Supporting Information.docx]

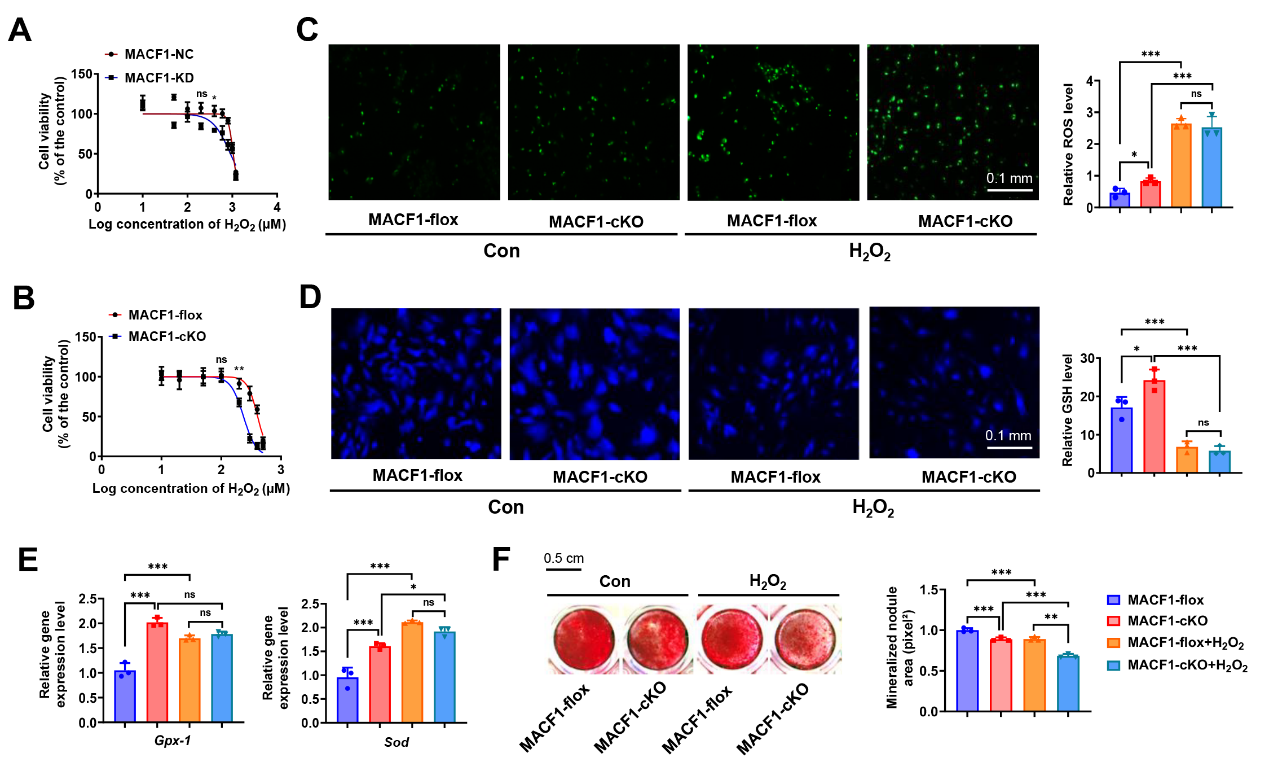


**S-Fig 1 MACF1 deficiency decreases the viability of osteoblasts treated with H_2_O_2_ and induces oxidative stress in primary cortical bone-derived mesenchymal stem cells (MSCs).** (A) Viability of MC3T3-E1 cells treated with 0, 10, 50, 100, 200, 400, 600, 800, 1000 or 1200 μM H_2_O_2_ for 48 h. (B) Primary MSCs treated with 0, 10, 20, 50, 100, 200, 300, 400 or 500 μM H_2_O_2_ for 48 h. MACF1-flox and MACF1-cKO MSCs were isolated from the bone tissue of MACF1-flox mice and MACF1 MSCs conditional knockout mice (MACF1-cKO). (C) ROS and (D) GSH levels in MACF1-flox and MACF1-cKO cells treated with or without 200 μM H_2_O_2_ for 12 h and quantitative analysis. Scale bars, 100 μm. (D) Sod and GPx-1 mRNA expression in MACF1-flox and MACF1-cKO cells treated with or without H_2_O_2_. (F) Representative Alizarin red S staining images and quantification analysis of MACF1-flox and MACF1-cKO cells treated with or without H_2_O_2_ and quantification analysis. Scale bars, 500 μm. **p*＜0.05, ** *p*＜0.01, *** *p*＜0.001.

**
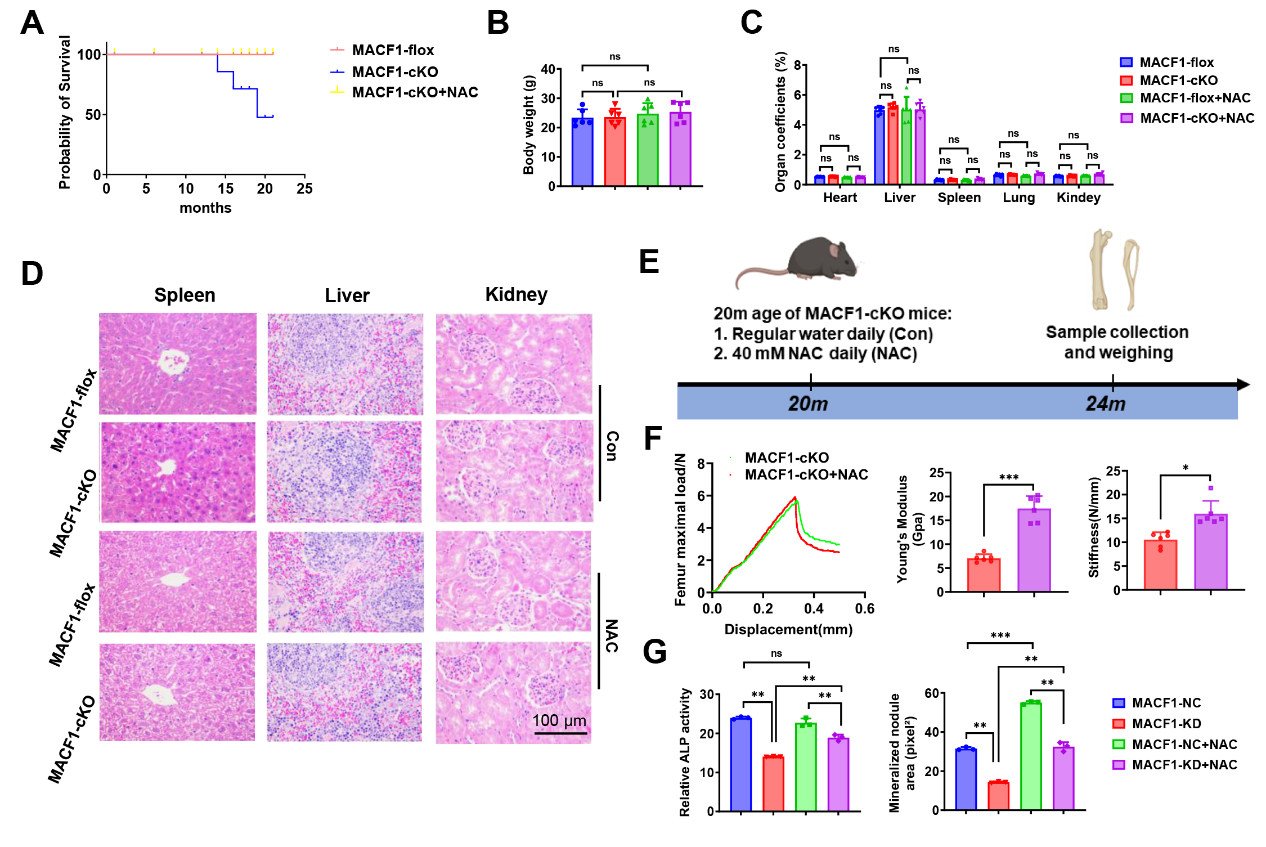
**

**S-Fig 2 NAC had no toxic effects on MACF1-cKO mice and enhanced the mechanical properties of the bone tissue in aged MACF1-cKO mice.** (A) Survival curve of MACF1-cKO mice treated with or without NAC, n=10 in each group. (B) Body weight and (C) organ coefficients of MACF1-flox and MACF1-cKO mice treated with or without NAC, n = 6 in each group. (D) Representative H&E staining images of the spleen, liver, and kidney of MACF1-flox and MACF1-cKO mice treated with or without NAC, n = 3 in each group; Scale bars, 100 μm. (E) Diagram depicting the administration of NAC treatment in MACF1-cKO mice from 20m to 24m of age. (F) Femur maximal load, Young's modulus, and stiffness of the tibia from aging MACF1-flox and MACF1-cKO mice determined using three-point bending, n = 6 in each group. (G) Quantification analysis of ALP activity and area of mineralized nodule. * *p*＜0.05, ** *p*＜0.01, *** *p*＜0.001.

**
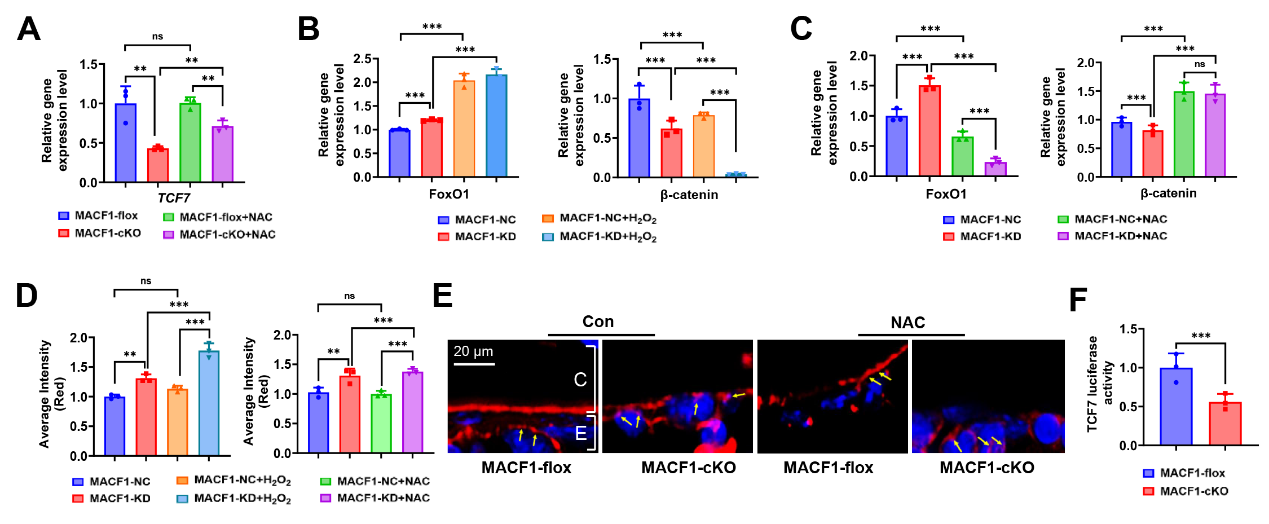
**

**S-Fig 3** **Quantitative analysis of the expression levels of FoxO1 and β-catenin, as well as the average intensity of FoxO1.** (A) The mRNA level of TCF7 in primary MSCs isolated form MACF1-cKO mice was detected following treatment with or without NAC. (B) The levels of FoxO1 and β-catenin in MACF1-NC and MACF1-KD cells were assessed following treatment with or without H_2_O_2_/(C) NAC. (D) The average intensity of FoxO1 was measured in MACF1-NC and MACF1-KD cells subjected to treatment with or without H_2_O_2_/NAC. (E) Representative immunofluorescence staining images of FoxO1 in bone tissue of MACF1-cKO mice treated with or without NAC. yellow arrow, FoxO1; C: cortical bone, E: endosteum. Scare bar: 20 μm. (F) Luciferase activity of TCF7 in primary MSCs isolated form bone tissue of MACF1-cKO mice. ** *p*＜0.01, *** *p*＜0.001.


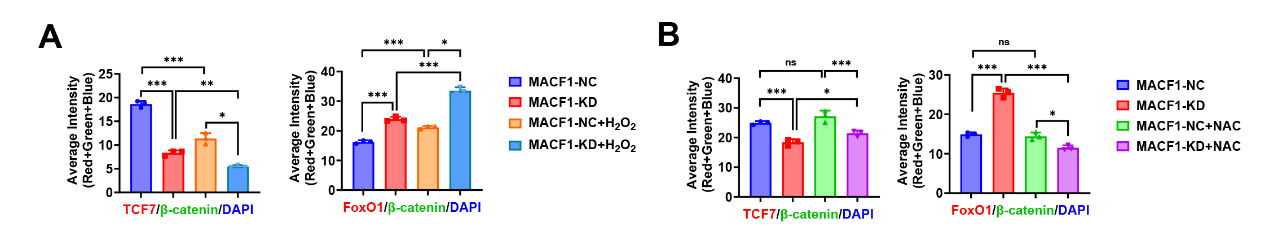


**S-Fig4 Quantitative analysis of** **colocalization.** (A) Colocalization of TCF7/β-catenin and FoxO1/β-catenin in MACF1-NC and MACF1-KD cells treated with or without H_2_O_2_/(B) NAC. * *p*＜0.05, ** *p*＜0.01, *** *p*＜0.001.
